# The Hagfish Gland Thread Cell: A Fiber-Producing Cell Involved in Predator Defense

**DOI:** 10.3390/cells5020025

**Published:** 2016-05-31

**Authors:** Douglas S. Fudge, Sarah Schorno

**Affiliations:** Department of Integrative Biology, University of Guelph, Guelph, ON N1G-2W1, Canada; sschorno@uoguelph.ca

**Keywords:** hagfish slime, gland thread cell, biomaterials, intermediate filaments, microtubules, nucleus, coiling, rotation

## Abstract

Fibers are ubiquitous in biology, and include tensile materials produced by specialized glands (such as silks), extracellular fibrils that reinforce exoskeletons and connective tissues (such as chitin and collagen), as well as intracellular filaments that make up the metazoan cytoskeleton (such as F-actin, microtubules, and intermediate filaments). Hagfish gland thread cells are unique in that they produce a high aspect ratio fiber from cytoskeletal building blocks within the confines of their cytoplasm. These threads are elaborately coiled into structures that readily unravel when they are ejected into seawater from the slime glands. In this review we summarize what is currently known about the structure and function of gland thread cells and we speculate about the mechanism that these cells use to produce a mechanically robust fiber that is almost one hundred thousand times longer than it is wide. We propose that a key feature of this mechanism involves the unidirectional rotation of the cell’s nucleus, which would serve to twist disorganized filaments into a coherent thread and impart a torsional stress on the thread that would both facilitate coiling and drive energetic unravelling in seawater.

## 1. Fibers in Biology

Fibrous structures, which are characterized by a high aspect ratio (*i.e.*, length/diameter), are ubiquitous in both engineering and biology. Fibers are useful for applications calling for high tensile strength, but low flexural rigidity. An example is a safety rope, which must bear significant tensional loads but also be flexible enough to coil. In biology, fibers are used for similar functions, such as spider draglines [1,2], and also are commonly found in fiber-reinforced biomaterials such as tendon and insect cuticle [3,4]. In these extracellular materials, stiff fibers (*i.e.*, collagen and chitin) are embedded in elastomeric matrices to form tough fiber-reinforced composites. Within eukaryotic cells, the best known structural fibers are the three main classes of cytoskeletal elements: F-actin, microtubules (MTs), and intermediate filaments (IFs), which participate in myriad cell functions, including cell division, cell motility, vesicular transport, and structural reinforcement.

The highest aspect ratio fibers in biology, which include the spider and insect silks, are produced extracellularly by specialized glands (Table 1). For example, the cocoon silk fiber spun by the silkworm moth larva (*Bombyx mori*) has a typical diameter of about 20 μm and a length of 1 km [5], for an impressive aspect ratio of 5 × 10^7^. In contrast, cytoskeletal elements in cells typically have aspect ratios of less than 10^3^. Neurofilaments (NFs), which are the IFs found within axons and dendrites, are an exception, and have been measured to have aspect ratios greater than 10^5^ in cultured cells [6], with values *in vivo* likely to be much larger.

## 2. Gland Thread Cells

**Figure 1:**
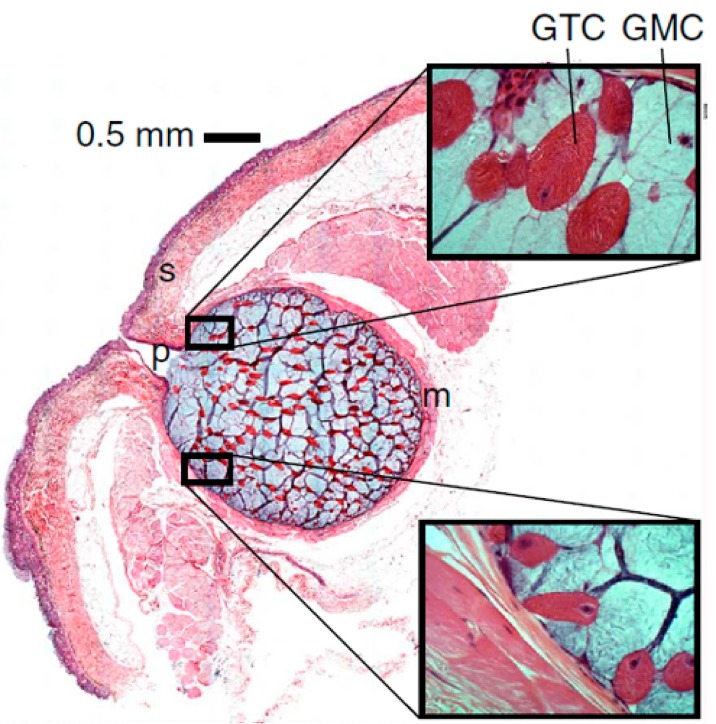
Histological cross-section through a slime gland. Hagfish slime glands, which are continuous with the skin (S), produce two main slime secretory cell types, the gland thread cells (GTCs) and gland mucus cells (GMCs). When provoked, the striated muscle around the gland (M) contracts, forcing the two cell types out through the gland pore (P) into the surrounding seawater, where they form into whole slime within a fraction of a second [24].

**Figure 2:**
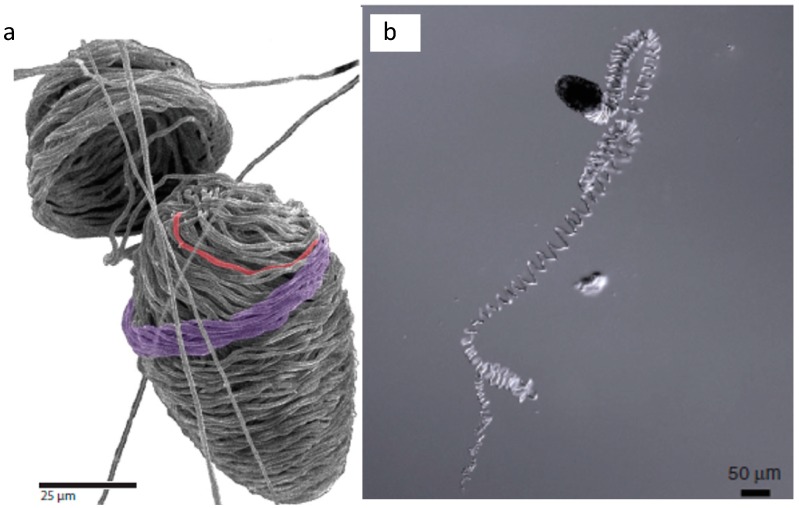
Skein structure and unravelling. (**a**) Scanning electron micrographs of a skin cracked open to reveal the internal organization of staggered thread loops [25]; (**b**) differential interference contrast micrograph of a partially untraveled thread skein [25].

The fiber-producing cells in hagfish slime glands (Figure 1), known as gland thread cells (GTCs) are unusual in that they produce a single protein fiber in their cytoplasm that is 1–3 μm in diameter and approximately 150 mm in length [18,20,21] for an aspect ratio of about 10^5^. This fact is all the more impressive given that the thread is not produced within an elongate structure such as an axon (as NFs are), but instead within an ellipsoid cell [18]. Within GTCs, the slime thread is produced from IFs and is elaborately coiled in a way that facilitates its unraveling when it is ejected from the slime gland along with mucus [22] into seawater (Figure 2). Hagfish slime threads are also unusual in that they are the only IF polymers that are destined for extracellular (and extracorporeal) use [23]. In all other known cases, IFs function within cells, whether as an integral part of the cytoskeleton in living cells, or as the fibrous reinforcement in the tough composites known as mammalian keratins, which are formed from the joining together of numerous keratinized cells [19].

As remarkable as the aspect ratio of hagfish slime threads is, it is likely not as high as it could be, given that they are built from 12-nm diameter IFs, with the mature threads having a diameter more than 100× greater than these constituent filaments [18,21]. It is therefore interesting to speculate about the selective pressures that have arrived at GTCs producing threads with their current dimensions. One of the primary functions of hagfish slime is to deter fish predators by clogging their gills [26,27]. The threads lend mechanical coherence to the slime, and also help prevent the predator from being able to flush the slime off via vigorous movements of the gill opercula. To perform this function, the threads need to be long enough to span adjacent gill filaments and arches and entangle with neighboring threads, with threads longer than this compromising thread diameter, and consequently the maximum tensile load each thread can bear. Perhaps thicker and adequately long threads are more effective at resisting the forces of gill flushing than the same number of long and skinny threads.

Another impressive aspect of GTCs is that the coiled thread (or skein) they produce unravels in a fraction of a second after being ejected from the slime gland [27]. The threads interact with secreted mucus (co-expressed from the slime glands; Figure 1) and seawater to form the mature defensive slime [23,19]. The unravelling of the threads is not fully understood, but it is clear that the coiling pattern of the thread within a GTC facilitates this rapid unravelling process and mitigates tangling and fouling of the thread [18,28]. Curiously, the skein unraveling process differs substantially in the two species of hagfish that have been investigated so far. In the Atlantic hagfish, *Myxine glutinosa*, unravelling is driven by external forces acting on the skein. Specifically, vigorous mixing and the presence of mucus are both required for unraveling to proceed [25]. In contrast, skeins from the Pacific hagfish, *Eptatretus stoutii*, do not require vigorous mixing or the presence of mucus; unraveling proceeds spontaneously, with loops of thread appearing to leap off the remaining coiled skein in the presence of seawater [23]. We recently showed that unraveling in *E. stoutii* involves the dissolution of a proteinaceous glue in seawater, and the subsequent release of strain energy stored in the thread [29]. This hypothesis is supported by the observation that unraveling can be induced using a protease under physical and chemical conditions where unravelling is normally inhibited (*i.e.*, low salt and elevated temperature). These results raise the interesting issue of what the source of the stored strain energy is and how it gets imparted to the thread.

## 3. Slime Thread Production in GTCs

**Figure 3:**
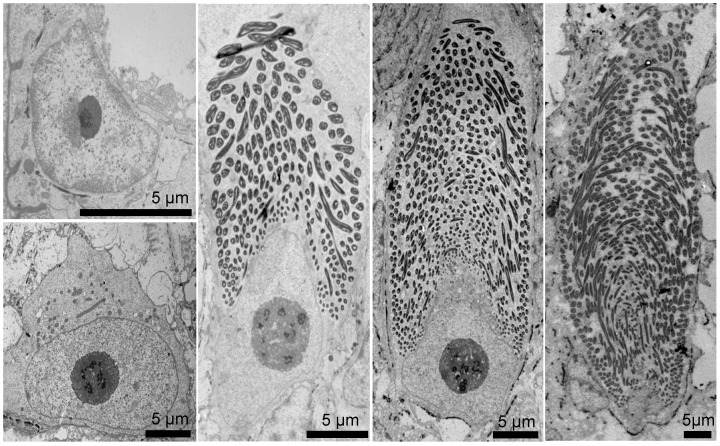
Transmission electron micrographs (TEM) of gland thread cell (GTC) development. Development series of hagfish GTCs reveals dramatic changes in nuclear size and shape as the cell matures. Immature GTCs, which lack a slime thread, can be identified by their prominent nuclei and nucleoli, while most of the space within a fully mature GTC is occupied by the thread skein. As the GTC develops, the nucleus becomes more spindle-like, eventually receding to the basal end of the cell in mature cells. Scale bars are 5 μm [24].

One of the main goals of our research is to understand how GTCs produce such a mechanically robust and high aspect ratio thread within the confines of their cytoplasm. At present, we do not have a comprehensive explanation, but microscopy of GTCs at various stages of development provides some clues (Figure 3). Histology of intact slime glands reveals that GTCs are present throughout the slime gland, and likely originate from stem cells at the gland epithelium [30,24]. Young GTCs begin their development with their longitudinal axis parallel to the epithelium, but as they grow and develop, their position shifts so that they ultimately are oriented with their longitudinal axis pointing toward the gland pore, through which skeins and mucous cell vesicles must pass to reach the external environment [22]. Mature GTCs are large cells, with a major axis length of about 125 μm. Immature GTCs near the gland epithelium are far smaller, with cells as small as 20 μm long exhibiting clear signs of a coiled thread [24]. Even younger GTCs, which can be identified before a thread is visible by their large nucleus with prominent nucleoli, can be smaller than 10 μm (Figure 3).

**Figure 4:**
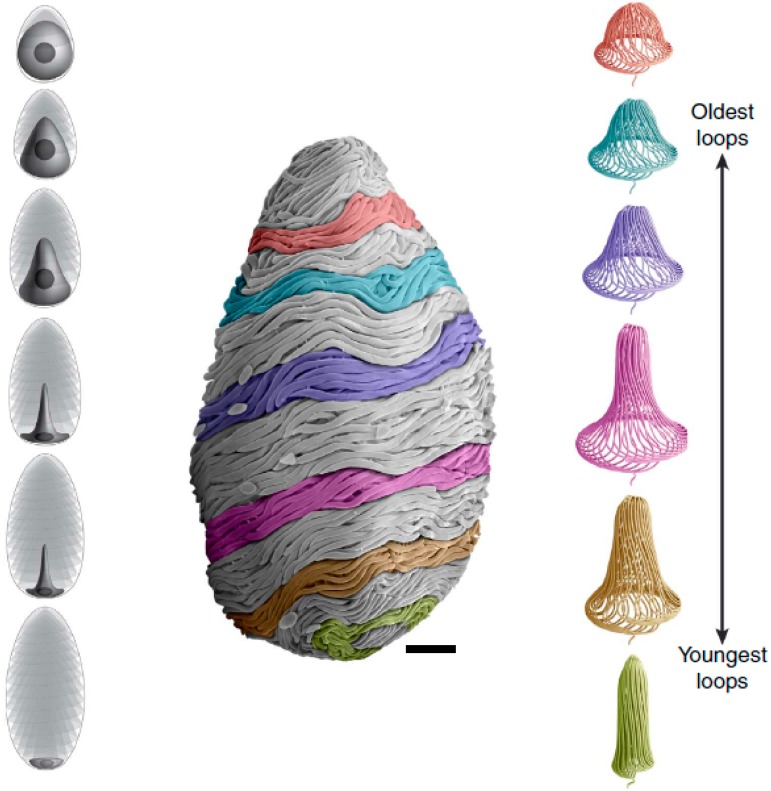
Developmental series of GTCs and thread coiling. (**Left**) The GTC nucleus in initially round in shape, occupying most of the space within the cell, but over time it takes on a spindle shape as more loops of thread are laid down, until the nucleus is just a disc at the basal end of a mature GTC. (**Right**) Successive arrangements of thread loops reflect the morphology of the nucleus at the time they were formed. (**Center**) The arrangement of the thread loops facilitates the rapid unravelling of the thread in secreted mature GTCs. Scare bar is 10 μm [24].

In mature GTCs, the thread is about 150 mm long and bi-directionally tapered, with a diameter in the middle of about 3 μm and closer to 1 μm at the two ends [18,19]. In the apical portion of the cell, there is no obvious coiling pattern, with the thread twisting and turning seemingly at random. In the rest of the skein, however, the thread is exquisitely coiled, with about 500 thread loops making up the most basic unit of organization [18,24,28]. These loops have a relatively tight bend at the apex of their trajectory, as well as ascending and descending segments that are connected by a circumferential run of about 60 degrees around the periphery of the cell. Each subsequent loop is staggered relative to its neighbor so that a complete 360-degree rotation of loops end up defining a conical structure that mirrors the morphology of the apical surface of the nucleus at the time it was formed (Figure 4). These conical loop arrangements spiral along the long axis of the cell for approximately 15–20 full rotations. At the skein surface, the nested arrangement of the circumferential runs of the loops gives the appearance that the thread is twisted into a cable; this, however, is an illusion created by the way the loops are packed at the skein periphery [18].

## 4. Thread Ultrastructure and IF Condensation

**Figure 5:**
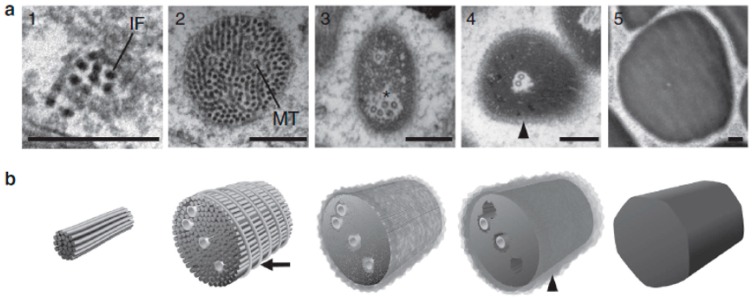
Changes in thread ultrastructure over gland thread cell (GTC) development. (**a**) Transmission electron micrographs (TEM) of thread cross-sections depicting changes in ultrastructure over development. The threads initially only consist of a bundle of intermediate filaments) (IFs) (1), but gradually increase in diameter via the addition of IFs and eventually microtubules (MTs) to the thread (2). The thread becomes more tightly packed with IFs around the MTs (3), until a fluffy rind appears on the thread surface (4). In fully mature thread, IF proteins are further compared, and the MTs and fluffy rind disappear from the thread, with the final product being electron-dense threads (5); (**b**) models illustrating the development of the thread as it matures, showing the transition from a bundle of a few IFs with intermediate MTs, to a fully condensed, mature thread [24].

Further clues about GTC development and thread maturation can be gleaned from TEM (Figure 5). In young GTCs, the thread appears as a simple parallel bundle of 12-nm IFs, with a diameter of about 30 μm [31]. In methylene blue stained plastic sections of young GTCs viewed with light miscroscopy, the early thread has a zigzag appearance as it coils in what appear to be multiple figure-eight curves [24]. Oddly, this zigzag pattern is not visible in TEM. The thread increases in diameter via the addition of more IFs, and eventually MTs appear in the thread interior [24,31]. At this stage, a 12-nm filament also becomes visible at the thread periphery. In longitudinal sections of the thread, this wrapping filament appears as regularly spaced dots, and in glancing sections, they can be seen travelling (nearly) orthogonally to the thread axis. It is postulated that these filaments are helically wound, but it is also possible that regularly spaced filament rings could result in the same patterns in TEM [24,31]. Later stages of GTC maturity are characterized by a dramatic shift in overall thread ultrastructure. Rather than appearing as distinct 12-nm filaments, IF proteins undergo a rearrangement that results with them condensing with their neighbors into a single super-fiber [20,24,31]. This condensation of IF proteins corresponds with the loss of the wrapping filament, and the appearance of a rind of material at the thread periphery that is distinctly less dense than the rest of the thread. MTs persist in the thread interior through the condensation event, but as the thread approaches its final stages of maturation and the flocculent rind disappears, MTs disappear and the space they occupied is filled in [20,24,31].

The ultrastructural changes in the thread described above raise several questions about thread growth and maturation. The role of the MTs in the thread is not known, although one possible function is to deliver IFs and/or IF subunits to the thread interior to increase the thread’s girth [24]. This hypothesis is supported by the fact that MTs can be seen penetrating the thread from the side, which is what they must do if they deliver building blocks from the cytoplasm to the thread interior. Another possibility is that the MTs provide structural support to the growing thread and impart flexural rigidity in a way that facilitates coiling. Another puzzle is the function of the wrapping filament. Downing *et al.* [31] suggested that it functions to regulate the addition of new IFs to the growing thread, but it is unclear how this would work, especially if, as they also suggest, IF are added to the thread periphery (thereby making the wrapping no longer peripheral). An alternative possibility is that secondary growth occurs via IFs being delivered to the thread interior via MTs, and the wrapping filament is a mechanism to prevent the merging of adjacent threads. If this is the case, it raises the question of how the wrapping filament accommodates increases in thread diameter.

IF condensation is not yet fully understood, although it is likely that it involves a modification of thread IF proteins that occurs globally and rapidly in the GTC, given the fact that the entire thread in all cells observed can be classified clearly as pre- or post-condensation. One possible explanation is that phosphorylation of thread proteins leads to a partial disassembly of thread IFs, and their reassembly into a single massive IF-like structure. Indeed, one of the constituent IF proteins in the thread, α, undergoes post-translational modifications (PTM) to become ß, which is the dominant isoform in mature GTCs [32]. It is likely that this PTM is phosphorylation, which is known to be involved in the disassembly of IF networks in other cells, most notably prior to mitosis [33]. One interesting aspect of IF condensation is that it does not represent the final stage of thread maturation, as the thread continues to increase in diameter after it occurs [24,31]. It is not clear at this time whether the length of the thread continues to increase after condensation is complete. While growth of the thread before condensation likely involves recruitment of mature 12-nm IFs, no mature IFs are visible near the thread in post-condensation cells. Instead, growth likely involves the addition of IF subunits or proteins directly to the thread [24,31]. This is consistent with the idea that condensation involves a rearrangement of IF proteins into a single super-IF. Furthermore, the flocculent rind around the thread may be a zone where IF proteins are becoming incorporated into the thread, but have not yet fully assembled and condensed. Further *in vitro* work on the assembly behavior of phosphorylated and de-phosphorylated slime thread proteins will be needed to understand this process more deeply.

## 5. Mechanism of Thread Coiling

One of the biggest remaining questions about GTCs is how they achieve such exquisite coiling of the thread. The regular pattern of staggered thread loops and spiraling conical loop arrangements is suggestive of a spinning mechanism within the cell [24]. Is it possible that the thread is formed via a process similar to a spinning wheel, in which disorganized textile fibers are twisted into a coherent and mechanically robust yarn? Such a wheel-like structure may exist in the cytoplasm near the apical side of the nucleus, but it seems unlikely that the several groups that have examined GTC ultrastructure would have missed it [20,24,31]. Another possibility is that the entire GTC nucleus rotates and acts as a spinning wheel that twists newly assembled IFs into a coherent slime thread.

## 6. The GTC as Spinning Wheel: The Nuclear Rotation Hypothesis

**Figure 6:**
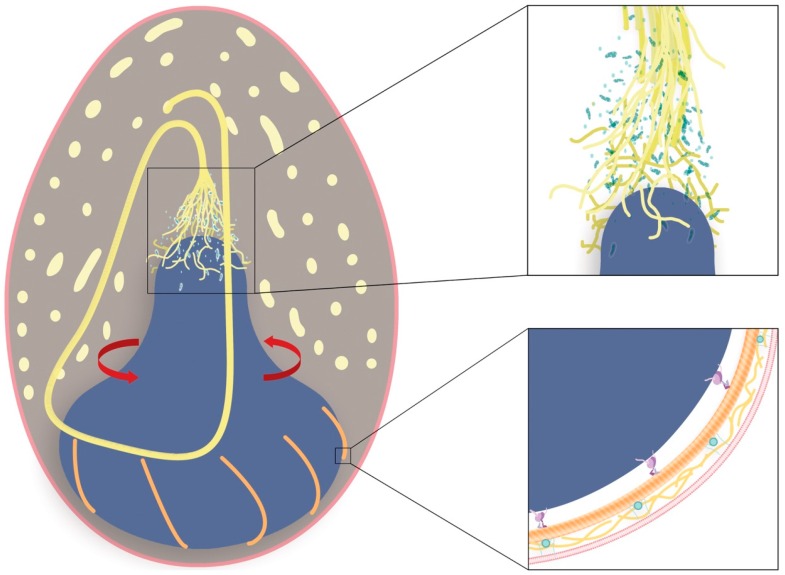
Nuclear rotation hypothesis. The nuclear rotation hypothesis explains how the thread becomes so neatly coiled, with IFs twisted together in to a coherent thread near the apical tip of the nucleus (dark blue), similar to how a spinning wheel twists wool fibers into yarn. The growing thread loops coil within the space defined by the apical surface of the nucleus and the already coiled thread loops (oblique and cross-sections shown in pale yellow). Inset top: IF proteins are synthesized and IFs (yellow) are assembled within the mitochondrial rich zone (MRZ) near the apical tip of the nucleus (green), which is rich in mitochondria and ribosomes (light blue). Inset bottom: Nuclear rotation powered by dynein molecules motors (purple), which process along MTs (orange) that spiral out form a microtubule organizing center at the base of the cell. We speculate that dynein is anchored to the nuclear envelope (green) via KASH protein domains, while the MTs are attached to the plasma membrane (pink) via interactions with formin protein (teal) and actin filaments (gold). Image credit: Jenny Moring and Julia Krolik (Art the Science Inc., Kingston, Canada).

The nuclear rotation hypothesis (Figure 6) is appealing in that it explains several puzzling aspects of thread formation in GTCs. Firstly, it explains how the thread becomes so neatly coiled. It is possible that the regular coiling is a result of a random event early in the life of the cell that then sets the direction of the subsequent passive coiling behavior. However, if this were the case, one might expect to see the occasional switching of coiling direction in a GTC, and we never do. Furthermore, we also see consistent handedness of coiling in all GTCs examined so far, which would not be the case if the direction of coiling were determined randomly. Having the thread produced on a spinning nucleus provides a mechanism for adding twist to the growing thread, which should have several benefits. One is that it should bias the direction of coiling and keep it consistent. Another is that it could add the twists that are necessary for forming the several hundred loops in the skein, which lie on top of each other like a rope that has been “flaked” on the deck of a boat for tangle-free deployment [28]. Anyone who has coiled a hose or rope knows that adding a twist for each loop makes the job far easier. Yet another advantage is that twisting the thread in the area of thread elongation could be an essential part of how newly synthesized and disorganized IFs are incorporated into the growing thread. Another appealing feature of this hypothesis is that it provides an explanation for the source of the strain energy that is released from the thread when the adhesive holding it together is dispersed by seawater or digested with trypsin. Namely, the strain energy released may be torsional energy imparted to the thread via nuclear rotation, with its ultimate source being the ATP-dependent walking of nucleus-tethered molecular motors along stationary MT tracks.

Wheels are extremely rare in biology [34], so we should be cautious about adopting this hypothesis before it has been adequately tested. How feasible is it that the GTC nucleus could rotate? Others have documented nuclear rotation in both healthy tissue (e.g., *Drosophila* embryos) [35] and as an aberrant behavior in cells expressing mutant versions of the intranuclear protein lamin A/C [36]. In both of these cases, nuclear rotation is not unidirectional, with the direction and speed exhibiting seemingly random fluctuations. In one study, rotation was found to be both ATP and dynein dependent, with the Type III IF vimentin acting as a brake on rotational movements [35]. In light of these findings, it is not difficult to imagine how nuclei in GTCs might have evolved not only to rotate, but to rotate consistently in one direction. We should point out that we are not the first to propose that nuclear rotation might be involved in the coiling of a cytoskeletal polymer—it was suggested that the twin nuclei in lamprey skein cells may revolve around each other, binary-star like, to produce the corkscrew appearance of the tonofilament bundles in those cells [37].

## 7. Details of Nuclear Rotation

If thread formation is driven by nuclear rotation, how might such a mechanism work? The model laid out in Figure 6 provides one possibility. Here, nuclear rotation is powered by the microtubule motor dynein, which has been implicated in nuclear positioning and movement in other studies [35,38,39,40,41,42]. Dynein is used to transport cargo around the cell by converting the chemical energy of ATP into retrograde movements along MT tracks. Dynein has been shown to associate with the outer nuclear membrane (ONM) via KASH (Klarsicht-ANC-1-SYNE homology) domain proteins, such as nesprin 1 and 2 in mammals or Klarsicht in *Drosophila* [43,44]. These KASH domain proteins are in turn tethered in the ONM by transluminal interactions with SUN domain proteins, which span the perinuclear space (PNS) into the inner nuclear membrane (INM) [45]. SUN-KASH proteins, commonly referred to as LINC (linker of the nucleoskeleton and cytoskeleton) complexes, form a molecular chain that spans both nuclear membranes and acts to mechanically couple nuclear and cytoplasmic structures [45,46,47,48]. If the nucleus acts as a spinning wheel, GTC nuclei are likely to be especially well endowed with lamin proteins, which are known to impart mechanical integrity to cell nuclei [49]. A robust lamin network inside the INM would help resist the shear forces exerted by dynein motors as they walk along immobilized MTs. If this is the case, then one would expect GTC nuclei to be stiffer than typical nuclei when probed with techniques such as micropipette aspiration [50,51].

**Figure 7:**
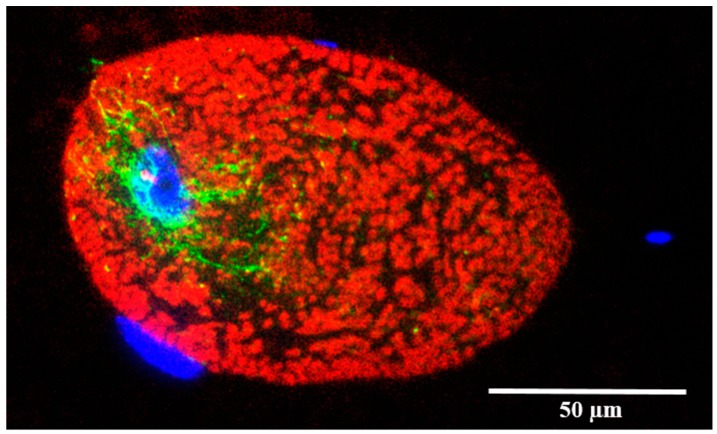
Epifluorescent image of an almost mature hagfish GTC in a fixed and paraffin-embedded slime gland. Fluorescently labelled IF (red) are abundant throughout the GTC cytoplasm, occupying most of the space within this mature GTC, while MTs (green) appear to be most numerous in the area around the cell’s nucleus (DAPI, blue). Nuclei of three other non-GTC cells can be seen in blue [52].

Generating nuclear spinning via the action of dynein motors would require an array of MTs anchored to an immobilized cell cortex, otherwise the forces exerted by dynein would result in translocation of the MTs instead of nuclear movements. The orientation of the MTs would also be critical, as anti-parallel MTs would result in opposing rotational forces acting on the nucleus. One possibility is that MTs radiate out from a microtubule organizing center (MTOC) at the base of the GTC, and wrap around the nucleus in a spiral pattern (Figure 6). The spiral pattern is critical, as perfectly radial MT spokes would result in the net force exerted by the dyneins being directed toward the basal end of the cell, given that dynein processes toward MT minus ends, which are anchored in MTOCs. If, however, MTs spiral around the nucleus with consistent handedness, the net force exerted on the nucleus will tend to keep the nucleus at the base of the cell, but will also have a rotational component (Figure 6, inset). Immunostaining of MTs reveal their presence on the basal side of the nucleus [52] (Figure 7). At the apex of the GTC nucleus sits a conical region known as the mitochondrial rich zone (MRZ), which contains both abundant mitochondria and ribosomes [31]. We propose that the MRZ is rigidly attached to the nucleus so that it rotates along with it. The region of cytoplasm just apical to the MRZ, however, is where IFs that have assembled from proteins synthesized in the MRZ may be twisted into a coherent thread. This is the enigmatic, and as yet undescribed, region of thread elongation. Detailed ultrastructure of this region in immature GTCs may provide vital clues about how thread elongation proceeds. It is worth noting that GTCs are conspicuously devoid of endoplasmic reticulum, which, if present, would likely weaken the shear strength of the linkage between the nucleus and the MRZ.

## 8. The Biomechanics of Thread Spinning via Nuclear Rotation

Although the nuclear rotation hypothesis has the potential to explain many aspects of thread production and coiling, it also raises new and interesting questions about how exactly this process might work and how it might have evolved. One pressing question is how the nascent thread is escorted away from the region of thread elongation instead of just piling up on the nuclear apex in a supercoiled tangle. In a traditional spinning wheel, newly formed yarn is taken up on a spool in a way that maintains constant tension on the thread and prevents it from recoiling back onto the spindle. Do GTCs possess such a structure and mechanism, and if so, what is it? One possibility is that the thread is guided away by growing MTs that have their minus ends anchored in the thread, and their growing ends impinging against something solid at the basal end of the cell. In this scenario, MTs could maintain tension in the thread and in so doing would become loaded in compression. Another possibility is that loops of thread guided away by MTs adhere to previously produced loops, perhaps via homophilic interactions of the wrapping filament. These kinds of interactions would have the added benefit of stabilizing the thread coils and preventing the torsional energy imparted to the thread from building up and feeding back to the region of thread elongation, which could ultimately bring nuclear rotation to a standstill.

It is also interesting to speculate about the mechanical design of a nuclear spinning wheel driven by dynein. We have not attempted to estimate the torque required to spin the nucleus and twist newly formed IFs into the nascent thread, but it is clear that it will depend on the number of dynein motors as well as their distance from the rotational axis. Positioning the motors at the widest part of the nucleus would maximize the mechanical advantage of the system, as torque is proportional to the product of the force exerted and the distance from the axis of rotation. Greater torque comes at the expense of slower angular velocity, but the fact that the base of the nucleus remains flared even at later developmental stages when the nucleus is mostly spindle-shaped (Figure 3, Figure 4 and Figure 6) suggests that maintaining torque might be favored over rotational velocity.

## 9. The Evolutionary Origins of GTCs

**Figure 8:**
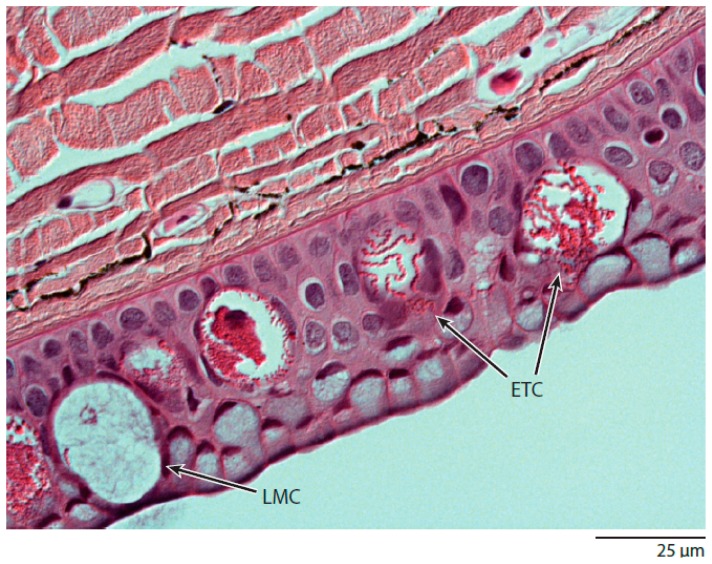
Histological cross-section through hagfish skin. Within the hagfish epidermis are two large secretory cell types, large mucus cells (LMCs) and epidermal thread cells (ETCs). These two epidermal cell types share similarities with the two main secretory cell types within slime glands, gland mucus cells (GMCs) and gland thread cells (GTCs). Both LMCs and GMCs are packed with mucin vesicles, while ETCs and GTCs both produce a coiled proteinaceous thread in their cytoplasm [55].

The evolutionary origin of GTCs is unclear, although hagfish skin offers some possible clues. One hypothesis for the origin for the slime glands is that they arose via a process of invagination of the skin, with subsequent specialization of secretory cells of the epidermis into GTCs and the mucus-producing gland mucous cells (GMCs). Hagfish skin possesses unique cells called epidermal thread cells (ETCs) (Figure 8), which possess coiled protein threads in their cytoplasm, although the threads are not nearly as long or organized as slime threads, and it appears that there may be more than one thread per cell [53]. Perhaps selection acted on ETCs after slime glands first appeared and resulted in an increase in the strength and aspect ratio of the threads they produce. The fact that the skin of hagfishes’ closest living relatives, the lampreys, possess ETC-like cells (called “skein cells”) also supports the hypothesis that the GTC lineage can be traced back to the epidermis [54].

## 10. Summary and Future Implications

In summary, hagfish GTCs are unique secretory cells that manufacture a high-aspect ratio protein thread in their cytoplasm using an as-yet undescribed mechanism. One possible mechanism is that disorganized cytoplasmic IFs are twisted into a coherent and neatly coiled slime thread via the rotation of the GTC nucleus. We propose that rotation is powered by dynein motors that are tethered to the widest part of the nucleus and walk on MTs firmly bound to the cell cortex. Testing this hypothesis will involve live cell imaging of immature GTCs and immunolocalization of MTs and dynein, as well as detailed ultrastructural analysis of the region of thread elongation. If nuclear rotation is found to occur in GTCs, it would raise the possibility that nuclear rotation could be functional in other cells. It could also provide a model for the construction of transgenic and/or cell free platforms engineered to produce high-performance protein threads *in vitro*.

## Figures and Tables

**Table 1:** Examples of fibers in biology, from lowest to highest aspect ratio.

Fiber	Average Length	Average Diameter	Aspect Ratio	References
Chitin fibrils	320 nm	3 nm	1.1 × 10^2^	[7]
Intermediate filaments (IFs)	1–2 μm	10 nm	2 × 10^2^	[8,9]
F-actin	1–2 μm	7 nm	2.8 × 10^2^	[10,11]
Mussel byssal threads	50 mm	0.1–0.2 mm	5 × 10^2^	[12]
Microtubules (MTs)	10–20 μm	25 nm	8 × 10^2^	[13,14]
Collagen fibrils	1 mm	50–500 nm	2 × 10^3^	[15]
Mammalian keratins (wool)	40–120 mm	10–20 μm	6 × 10^3^	[16,17]
Neurofilaments (NFs)	100 μm	10 nm	1 × 10^4^	[6]
Hagfish slime threads	150 mm	2 μm	7.5 × 10^4^	[18,19]
Spider (dragline) silk	30–60 m	1–5 μm	1.5 × 10^7^	[1,2]
Silkworm (*Bombyx mori*) silk	1 km	20 μm	5 × 10^7^	[5]
